# Boolean Combinations of Implicit Functions for Model Clipping in Computer-Assisted Surgical Planning

**DOI:** 10.1371/journal.pone.0145987

**Published:** 2016-01-11

**Authors:** Qiqin Zhan, Xiaojun Chen

**Affiliations:** School of Mechanical Engineering, Shanghai Jiao Tong University, Shanghai, China; North Shore Long Island Jewish Health System, UNITED STATES

## Abstract

This paper proposes an interactive method of model clipping for computer-assisted surgical planning. The model is separated by a data filter that is defined by the implicit function of the clipping path. Being interactive to surgeons, the clipping path that is composed of the plane widgets can be manually repositioned along the desirable presurgical path, which means that surgeons can produce any accurate shape of the clipped model. The implicit function is acquired through a recursive algorithm based on the Boolean combinations (including Boolean union and Boolean intersection) of a series of plane widgets’ implicit functions. The algorithm is evaluated as highly efficient because the best time performance of the algorithm is linear, which applies to most of the cases in the computer-assisted surgical planning. Based on the above stated algorithm, a user-friendly module named SmartModelClip is developed on the basis of Slicer platform and VTK. A number of arbitrary clipping paths have been tested. Experimental results of presurgical planning for three types of Le Fort fractures and for tumor removal demonstrate the high reliability and efficiency of our recursive algorithm and robustness of the module.

## Introduction

Computer-assisted surgical planning, a technique integrating the computer technology in presurgical planning and navigation, has been used to improve the safety and accuracy of the surgery [1], [2]. Orthopaedic surgical planning is a typical example. It provides surgeons with templates of orthopaedic prostheses [3]. With the representing of the patients’ image, surgeons can reconfigure geometrical constructs to match the anatomical features shown in the image and then the most suitable parameters of the templates of orthopaedic prosthesis for the patients are selected [4]. During the process, surgeons perform manipulations such as rotation, zooming, measurement and clipping of the models in the surgical planning softwares. Among these manipulations, model clipping is of great importance because surgeons need to find a appropriate and convenient clipping path to separate those fractured bones before taking the real operation.

Many softwares have been developed for computer-assisted surgical planning. For example, Magic Rapid Prototyping(or Magic RP), used for 3D imaging reconstruction [5], [6], is served as presurgical planning for osteotomy, including the manipulation of clipping and repositioning those fractured bones. A polyline served as the clipping path is generated based on the fiducial points created on the model surface. Although this polyline is extruded when clipping the model, the clipping path is actually two-dimensional and the polyline is same on the third dimension, which limits the contour of the clipped models on this dimension. Moreover, Magic RP can only cut through the whole object instead of cutting the desirable part with certain thickness, which is a disadvantage for clinical practice. Digital tools provided by TraumaCad, including an extensive digital template library and a full set of wizards and measurement tools, can be used to perform preoperative planning and allows orthopedic surgeons to simulate the expected results prior to surgery [7]. OrthoView is chosen by orthopaedic surgeons worldwide to create detailed pre-operative plans quickly and easily from digital x-ray images [8]. However, the manipulations of these similar softwares are based on the anteroposterior and lateral films, which makes the visualization not so friendly. In addition, these softwares are not open-source.

Our research is related to the work of both mesh segmentation and polygon clipping algorithms in computer graphics. Firstly, a number of algorithms for mesh segmentation have been proposed to separate the semantically meaningful regions, such as the method of K-mean clustering [9], spectral clustering methods [10], region growing [11], random walk algorithm [12], the primitive-fitting-based method [13] and so on. These algorithms are based on minima rule suggested by Hoffman [14] and on Watershed algorithm that is extended from image processing to 3D mesh segmentation [15]. Some algorithms achieve good results in partitioning, however, their algorithms also have limitations to apply for all kinds of models. In some cases, their algorithms may fail to produce the segmentation that is consistent to human perception and they have to manually adjust the parameters to achieve desirable segmentations [16]. Some others such as the method mentioned in [17] may result in under-segmentation or over-segmentation for specific models. This procedure is time-consuming and exhaustive. What’s more, their approaches may not be suitable for computer-assisted surgical planning because the clipping boundary is required to be along the specified path based on the clinical demand rather than human perception. Our module makes the presurgical planning path interactive during its construction.

Secondly, several polygon clipping algorithms are developed to visualize the model inside the clipping window. Such efforts include Sutherland-Hodgman algorithm, Weiler-Atherton clipping algorithm, Vatti clipping algorithm and so on. Sutherland-Hodgman algorithm obtains the intersection of the subject polygon and clip polygon [18] [19] by extending in turn the each line of clip polygon and choosing the vertices on the subject polygon that are on the visible side. Weiler-Atherton clipping algorithm works by recursively subdividing the image into polygon shaped windows until the depth order within the window is found [20]. However, these two algorithms are more suitable for clipping convex polygons. Vatti clipping algorithm does not restrict the type of subject polygon and clip polygon and can even be used to clip self-intersecting polygon with holes [21]. But this method is generally applicable only in 2D space [22]. In contrast, our clipping algorithm works well with both the convex and concave cases of model clipping in 3D space by means of implicit function that encloses the subject model. The remaining part of this paper manly deals with the algorithm how to obtain that implicit function through Boolean combination of the clipping path.

In this paper, an interactive method is presented for surgeons to make computer-assisted surgical planning. A module named SmartModelClip is developed on the basis of 3D-slicer [23], leveraging the classes and functions provided by VTK [24]. The clipping path in our module, composed of a series of connected plane widgets, can be adjusted to create desirable clipping path. A plane widget is a quadrilateral with handles on the vertexes to adjust the widget’s shape and a normal handle in the center to adjust the widgets’ position, which will make the presurgical planning interactive and convenient. A thickness plane widget is constructed to clip the model with certain thickness. After the construction of the whole clipping path, a recursive algorithm is implemented to acquire the implicit function of the clipping path. The algorithm is based on the Boolean combinations of the plane widgets’ implicit functions. The implicit function is served as data filter to separate any part of the model that should be clipped away. The model is finally separated by the class of ‘vtkClipPolyData’ [25] in VTK using the data filter. The robustness and performance of the algorithm have been extensively evaluated on our module with a great number of clipping paths and models.

This paper is organized as follows: In section 2,we describe the recursive algorithm for resolving the implicit function and provide three different types of examples to implement the algorithm. In section 3, we develop a module named SmartModelClip and analyze the robustness of the algorithm. In section 4, we discuss the time performance of the clipping algorithm and compare it with some other polygon clipping algorithms. Our mesh segmentation method is then compared with other methods in terms of segmentation time. And finally conclusions are made in the last section.

## Materials and Methods

Our approach to the acquirement of the clipping path’s implicit function is on the basis of a recursive algorithm. The recursive algorithm decomposes the problem into three subproblems according to the relative poses of plane widgets. The subproblems are combined through Boolean operations. The kind of Boolean operations is determined by the pose of each line segment of polyline on the transection plane of clipping path.

### Boolean Operations

**Fig 1:**
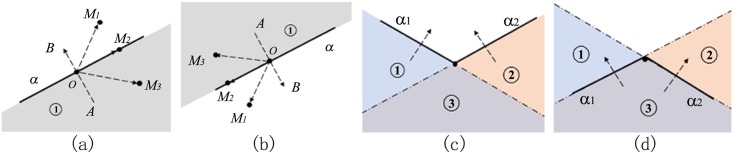
Boolean operations. (a)transection view of a plane widget *α* (region① is inside), (b)rotation of the plane widget in Fig 1(a)(region① is inside) (c)Boolean union of *α*_1_ and *α*_2_(region①, ② ③ are all inside), (d)Boolean intersection of *α*_1_ and *α*_2_(only region③ is inside).

Some notions about implicit function is first introduced. A plane widget is on the plane defined by the implicit equation of *F*(*x*, *y*, *z*) = 0, which separates the 3D space into three regions: on the plane(*F*(*x*, *y*, *z*) = 0), the region inside the plane(*F*(*x*, *y*, *z*)<0) and the region outside the plane(*F*(*x*, *y*, *z*)>0). A plane widget has the handle of normal axis fixed on the center of the plane widget, such as the handle **A** **B** in Fig 1(a).

Seeing from Fig 1(a), the judgement of a point position(outside, on or inside the plane widget) relies on the dot product of vector **A** **B** and vector **O** **M**_**i**_(*i* = 1, 2, 3):

$$AB\cdot OM_{i}=\{\begin{array}{rrr} F\left(M_{1}\right)>0 & \text{for}\;i=1\&\;M_{1} & \text{outside,}\\ F\left(M_{2}\right)=0 & \;\text{for}\;i=2\&\;M_{2} & \;\text{on},\\ F\left(M_{3}\right)<0 & \;\text{for}\;i=3\&\;M_{3} & \;\text{inside}. \end{array}$$ (1)

Comparing with Fig 1(a), the plane widget in Fig 1(b) is rotated in the 3D space and the Eq 1 is still valid. What’s different is that the inside region is at the top of the figure because the direction of the normal axis has been reversed. It’s clear that the region inside the plane is equivalent to the side of region where the initial point of the normal axis locates.

Two kinds of Boolean operations are used in the recursive algorithm to combine the implicit functions: Boolean union and Boolean intersection. Boolean union takes the minimum value of all implicit functions while Boolean intersection takes the maximum value of all implicit functions. For the concision of expression, Boolean union and Boolean intersection are denoted respectively by the commonly used notation of ∪ and ∩ in the set theory. To be specific, *α*_1_ ∪ *α*_2_ means the Boolean union of the implicit function of plane widget *α*_1_ and that of *α*_2_, while *α*_1_∩*α*_2_ means the Boolean intersection of the implicit function of plane widget *α*_1_ and that of *α*_2_. Fig 1(c) and 1(d) illustrate the result of *α*_1_∪*α*_2_ and *α*_1_∩*α*_2_. In Fig 1(c), region ① and region ② are inside *α*_1_, while region ② and region ③ are inside *α*_2_. After the Boolean operation of *α*_1_∪*α*_2_, all the colored regions(region ①, region ② and ③) are inside *α*_1_ *α*_2_. In Fig 1(d), region ② and region ③ are inside *α*_1_, while region ① and region ② are inside *α*_2_. The Boolean operation of *α*_1_∩*α*_2_ makes the region ③ inside the *α*_1_ *α*_2_.

It is noticed that the result of the Boolean operations of two implicit function is still an implicit function that the Boolean operations can still be applied to.

### Conversion of 3D Problem to 2D Problem

The problem of splitting 3D space by clipping path can be de degraded into the equivalent problem of splitting 2D space by a polyline on the transection plane(perpendicular to the first and second plane widget) of the clipping path. Thus the algorithm of acquiring the clipping path’s implicit function is implemented by judging the relative pose of the line segments. These line segments determine what kind of Boolean operations applies to the implicit function of each plane widget.

**Fig 2:**
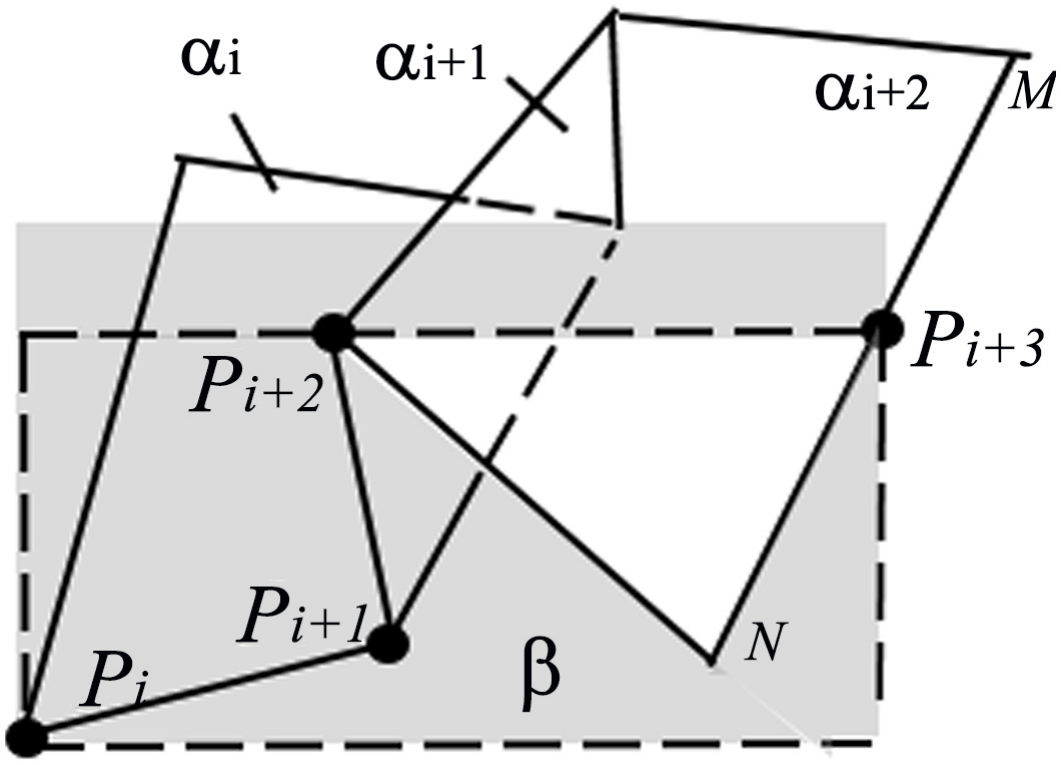
Three of the plane widgets *α*_*i*_*α*_*i*+1_*α*_*i*+2_ being transected by plane *β*. *β* is perpendicular to the plane widget *α*_1_ and *α*_2_.

**Fig 3:**
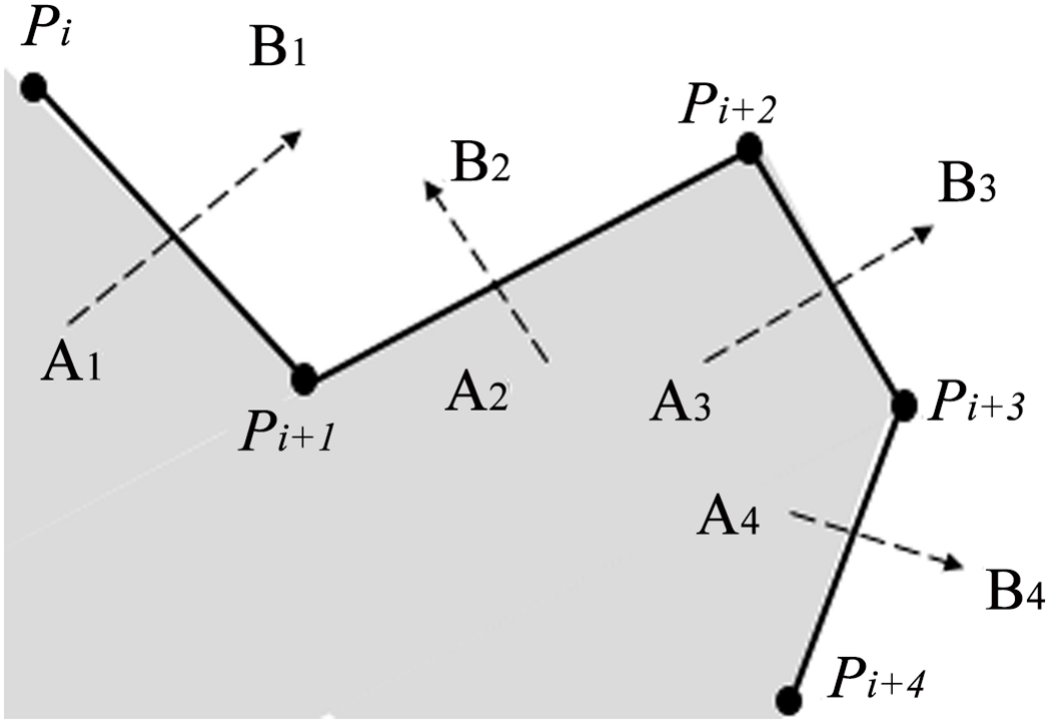
Directions of the normal axes of the plane widgets.

What is important when implementing the algorithm is whether a plane widget is inside or outside its adjacent plane widget. It determines whether to apply the Boolean union or the Boolean intersection. Fig 2 takes out three plane widgets from the clipping path that are transected by the plane *β*, forming a polyline *P*_*i*_*P*_*i*+1_*P*_*i*+2_*P*_*i*+3_ that’s on the edge of *α*_*i*_*α*_*i*+1_*α*_*i*+2_. The transection plane is specified to be perpendicular to the plane widget *α*_1_ and *α*_2_(not drawn in Fig 2). What the recursive algorithm cares about is whether *P*_*i*+2_ is inside or outside line segment *P*_*i*_*P*_*i*+1_ and whether *P*_*i*+3_ is inside or outside line segment *P*_*i*+1_*P*_*i*+2_ and so on. To be more specific in Fig 3, *P*_*i*+2_ is outside line *P*_*i*_*P*_*i*+1_, *P*_*i*+3_ is inside line *P*_*i*+1_*P*_*i*+2_, *P*_*i*+4_ is inside line *P*_*i*+2_*P*_*i*+3_. With these information, the pose of each line segments of the polyline is identified to judge the type of Boolean operations applied to the implicit function of each plane widget. The judgement of the Boolean operations will be discussed in the section 3.3. In that case, the problem has been degraded.

The normal axes of plane widget series are arranged in a way that all the initial points of the plane widgets’ normal axes belong to one side of the polyline and the endpoints belong to the other. In Fig 3, four initial points(A1, A2, A3, A4) of normal axes are all inside the shaded region, while the endpoints(B1, B2, B3, B4) are all outside the shaded region.

### Recursive Algorithm

#### Boolean Combinations of the Implicit Functions

The combinations of the Implicit functions are based on the recursive algorithm, which decomposes the recursive problem into three subproblems. Subproblem1 acquires the implicit function of a plane widget. Subproblem2 combines the implicit functions of two adjacent plane widgets. Subproblem3 is the recursive problem itself, i.e., the problem of combining the implicit functions of a series of adjacent plane widgets. These three subproblems corresponds to three conditional branches in our algorithm. Each subproblem is easier to solve because it involves fewer implicit functions to combine and then the subproblem is decomposed again until the problem is finally simplified into subproblem1 or subproblem2 that involve only one or two plane widgets’ implicit functions to solve.

Some notations of the algorithm should be clarified before explaining the recursive algorithm. After the transection of the clipping path, a polyline *P*_*i*_ *P*_*i*+1_ ⋯ *P*_*j*_ *P*_*j*+1_ is on the edge of the connected plane widgets of *α*_*i*_*α*_*i*+1_…*α*_*j*_. To be more specific, line section *P*_*i*_ *P*_*i*+1_ is on the edge of *α*_*i*_, *P*_*i*+1_*P*_*i*+2_ is on the edge of *α*_*i*+1_, ⋯, *P*_*j*_ *P*_*j*+1_ is on the edge of *α*_*j*_.

**Algorithm** Recursive algorithm for Boolean combinations

**input:** the implicit function of *α*_*i*_ and that of *α*_*j*_

**output**: the combined implicit function of *α*_*i*_*α*_*i*+1_ ⋯ *α*_*j*_

1: **function** CombImpFuns(*α*_*i*_, *α*_*j*_)

2:  **if** *i* = = *j* **then**

3:   return *α*_*i*_

4:  **else if** (*j* − *i*) == 1 **then**

5:   **if** *P*_*j*+1_ inside *α*_*i*_ **then**

6:    return *α*_*i*_⋂*α*_*j*_

7:   **else**

8:    return *α*_*i*_⋃*α*_*j*_

9:   **end if**

10:  **else**

11:   *k* ← *j*

12:   **while** *Loop Condition*(@*line*26*th*) **do**

13:    *k* ← *k* − 1

14:   **end while**

15:   **if** *P*_*k*+1_ inside *α*_*k*−1_ **then**

16:    *CombinedFun* ← CombImpFuns(*α*_*i*_, *α*_*k*−1_)

17:          ⋂ CombImpFuns(*α*_*k*_, *α*_*j*_)

18:   **else**

19:    *CombinedFun* ← CombImpFuns(*α*_*i*_, *α*_*k*−1_)

20:          ⋃ CombImpFuns(*α*_*k*_, *α*_*j*_)

21:   **end if**

22:   return *CombinedFun*

23:  **end if**

24: **end function**

25:

26: *Loop Condition*:

27: line *P*_*k*_*P*_*k*+1_ and polyline ${P_{i}}^{\prime }P_{i}P_{i+1}$ ⋯ *P*_*k*−1_

28: intersect(for *i* + 2 ⩽ *k* ⩽ j)

29: **or** ray *P*_*k*−1_ *P*_*k*_ and polyline $P_{k+2}P_{k+3}\cdots P_{j}P_{j+1}P_{j+1}^{\prime }$

30: intersect (for *i* + 2 ⩽ *k* ⩽ *j* − 2)

Subproblem1 returns the implicit function of a plane widget.

Subproblem2 combines the implicit functions of two adjacent plane widgets as illustrated in Fig 1(c) and 1(d). It is noticed that as a result of *j* − *i* = 1, the point *P*_*j*+1_ is just the point *P*_*i*+2_. If the point *P*_*i*+2_ is inside plane widget *α*_*i*_, the algorithm applies the Boolean operation of *α*_*i*_ ∩ *α*_*i*+1_ or else it applies the Boolean operation of *α*_*i*_ ∪ *α*_*i*+1_.

Subproblem3 first extends respectively line segment of *P*_*i*_*P*_*i*+1_ and that of *P*_*j*_*P*_*j*+1_ to infinity on the direction of vector **P**_**i**+**1**_**P**_**i**_ and vector **P**_**j**_**P**_**j**+**1**_ and we get the ray $P_{i+1}{P_{i}}^{\prime }$ and the ray $P_{j}{P_{j+1}}^{\prime }$. And then the line segment of *P*_*k*_*P*_*k*+1_(*i* + 2 ⩽ *k* ⩽ *j*) is extended to infinitive on the both directions. *k* is initialized with the value of *j*. For the while-loop at line 12th, we check respectively whether line *P*_*k*_*P*_*k*+1_(*i* + 2 ⩽ *k* ⩽ *j*) and polyline ${P_{i}}^{\prime }P_{i}P_{i+1}$ ⋯ *P*_*k*−1_ intersect or the ray *P*_*k*−1_ *P*_*k*_(*i* + 2 ⩽ *k* ⩽ *j* − 2) and polyline $P_{k+2}P_{k+3}\cdots P_{j}P_{j+1}{P_{j+1}}^{\prime }$ intersect. If it does, the value of *k* is declined by 1. The while-loop is broken if any one of the conditions is satisfied: (1)*k* < *i* + 2, (2)line *P*_*k*_*P*_*k*+1_ and polyline ${P_{i}}^{\prime }P_{i}P_{i+1}\cdots P_{k-1}$ do not intersect, (3)ray *P*_*k*−1_*P*_*k*_ and polyline $P_{k+2}P_{k+3}\cdots P_{j}P_{j+1}P_{j+1}^{\prime }$ do not intersect. Finally, we check the relative pose of *α*_*k*−1_ and *α*_*k*_. If point *P*_*k*+1_ on the plane *α*_*k*_ is inside the plane widget *α*_*k*−1_, we apply the Boolean operation of *α*_*i*_*α*_*i*+1_ ⋯ *α*_*k*−1_ ∩ *α*_*k*_*α*_*k*+1_ ⋯ *α*_*j*_. Or else we apply the Boolean operation of *α*_*i*_*α*_*i*+1_ ⋯ *α*_*k*−1_ ∪ *α*_*k*_*α*_*k*+1_ ⋯ *α*_*j*_. The same algorithm applies to the implicit function of *α*_*i*_*α*_*i*+1_ ⋯ *α*_*k*−1_ and that of *α*_*k*_*α*_*k*+1_ ⋯ *α*_*j*_. In this way, the problem has been decomposed and will finally be simplified into the subproblem1 and subproblem2.

#### Examples for Implementing the Algorithm

Three different types of examples that implement the algorithm are given below to have a better understanding of the whole algorithm. Specifically, the while loop condition(line 26th to 30th of the clipping algorithm) is explained in detail. Although all the examples contain 4 plane widgets, the poses of the plane widgets are slightly distinct and the implementation process is totally different.

**Fig 4:**
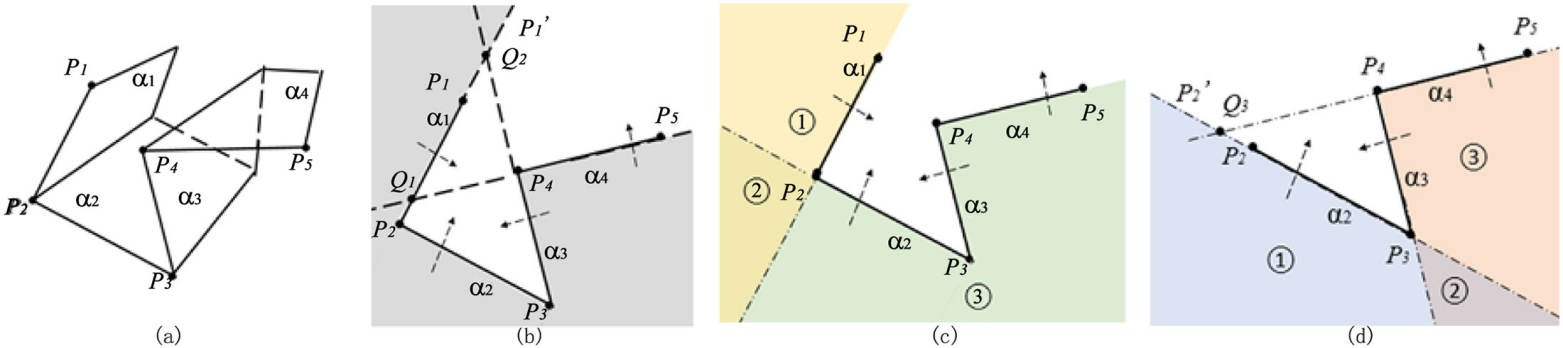
Implementation of algorithm for example1. (a)Clipping path with four plane widgets, (b)Transection view of clipping path, (c)Decomposition of the implicit function of *α*_1_*α*_2_*α*_3_*α*_4_ into that of *α*_1_ and that of *α*_2_*α*_3_*α*_4_, (d)Decomposition of the implicit function of *α*_2_*α*_3_*α*_4_ into that of *α*_2_ and that of *α*_3_*α*_4_.

For the first example in Fig 4(a), the clipping path is composed of four plane widgets which are transected by a plane that is perpendicular to plane widget *α*_1_ and plane widget *α*_2_, leaving the polyline *P*_1_*P*_2_*P*_3_*P*_4_*P*_5_ on the edge. The shaded space in Fig 4(b) is inside the implicit function of *α*_1_*α*_2_*α*_3_*α*_4_.

To get the implicit function of *α*_1_ *α*_2_ *α*_3_ *α*_4_, line segment *P*_2_*P*_1_ in Fig 4(b) is first extended on the direction of vector **P**_**2**_ **P**_**1**_ and it becomes the ray $P_{2}{P_{1}}^{\prime }$. And the line *P*_4_*P*_5_ is extended to infinite on both directions. Since line *P*_4_*P*_5_ and polyline ${P_{1}}^{\prime }P_{1}P_{2}P_{3}$ intersect at point *Q*_1_, the value of *k*(3 = *i* + 2 ⩽ *k* ⩽ *j* = 4) should be decreased to 3. Then we check whether line *P*_3_*P*_4_ and polyline ${P_{1}}^{\prime }P_{1}P_{2}$ (more precisely the ray $P_{2}P_{1}{P_{1}}^{\prime }$) intersect or not and they actually intersect at *Q*_2_. Therefore, the value of *k* should be decreased again to be 2. As the required minimum value of *k* is 3, seeing from line 28th of the algorithm, the loop condition is not satisfied after this decrement of *k*. *P*_3_ is outside plane widget *α*_1_, so it’s easy to get the implicit function of *α*_1_*α*_2_*α*_3_*α*_4_ on condition that the implicit function of *α*_1_ and that of *α*_2_*α*_3_*α*_4_ are known respectively because the implicit function of *α*_1_*α*_2_*α*_3_*α*_4_ is the result of *α*_1_ ∪ *α*_2_*α*_3_*α*_4_. Region ① and region ② are inside the implicit function of *α*_1_, while region ② and region ③ are inside the implicit function of *α*_2_*α*_3_*α*_4_ in Fig 4(c). Boolean operation of *α*_1_ ∪ *α*_2_*α*_3_*α*_4_ makes the regions of ①, ② and ③ inside the implicit function of *α*_1_*α*_2_*α*_3_*α*_4_.

Now that the implicit function of *α*_1_ is the solution of subprolem1, the task is to get the implicit function of *α*_2_*α*_3_*α*_4_. As *P*_4_*P*_5_ and extended polyline ${P_{2}}^{\prime }P_{2}P_{3}$ intersect at *Q*_3_ in Fig 4(d), *k*(4 = *i* + 2 ⩽ *k* ⩽ *j* = 4) is decreased to 3. So it comes out of the loop for *k* = 3. As *P*_4_ is outside plane widget *α*_2_, the implicit function of *α*_2_*α*_3_*α*_4_ is available through the Boolean operation of *α*_2_ ∪ *α*_3_*α*_4_. Region ① and region ② are inside the implicit function of *α*_2_, while region ② and region ③ are inside the implicit function of *α*_3_*α*_4_ in Fig 4(*d*). Boolean operation of *α*_2_ ∪ *α*_3_*α*_4_ makes the regions of ①,② and ③ inside the implicit function of *α*_2_*α*_3_*α*_4_.

The implicit function of *α*_2_ is the solution of subproblem1 and the implicit function of *α*_3_ *α*_4_ is the solution of subproblem2, so the whole problem is solved by the Boolean combinations of implicit functions of all the plane widgets. In conclusion, the implicit function of *α*_1_*α*_2_*α*_3_*α*_4_ is available through the following equation:

$$\begin{array}{cc} \alpha_{1}\alpha_{2}\alpha_{3}\alpha_{4} & =\alpha_{1}\cup \left(\alpha_{2}\alpha_{3}\alpha_{4}\right)\\ & =\alpha_{1}\cup \left(\alpha_{2}\cup \left(\alpha_{3}\alpha_{4}\right)\right)\\ & =\alpha_{1}\cup \left(\alpha_{2}\cup \left(\alpha_{3}\cap \alpha_{4}\right)\right). \end{array}$$ (2)

**Fig 5:**
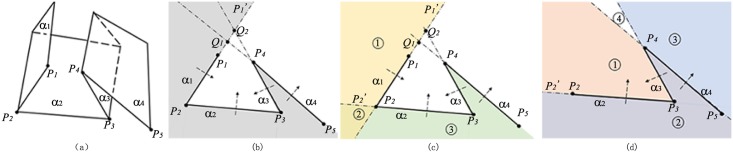
Implementation of algorithm for example2. (a)Clipping path with four plane widgets, (b)Transection view of clipping path, (c)Decomposition of the implicit function of *α*_1_*α*_2_*α*_3_*α*_4_ into that of *α*_1_ and that of *α*_2_*α*_3_*α*_4_, (d)Decomposition of the implicit function of *α*_2_*α*_3_*α*_4_ into that of *α*_2_*α*_3_ and that of *α*_4_.

For the second example in Fig 5(a), the poses of this clipping path is different from those of the Example 1 only in the relative pose of *α*_4_ to that of *α*_3_, from which we’ll see that different poses of plane widgets lead to different expressions of the implicit function yet using the same recursive algorithm.

Line *P*_4_*P*_5_ and polyline ${P_{1}}^{\prime }P_{1}P_{2}P_{3}$ intersect at *Q*_1_; line *P*_3_*P*_4_ and polyline ${P_{1}}^{\prime }P_{1}P_{2}$ at *Q*_2_ in Fig 5(b), so the way to acquire the implicit function of *α*_1_*α*_2_*α*_3_*α*_4_ is to apply the Boolean operation of *α*_1_ ∪ *α*_2_*α*_3_*α*_4_ as is illustrated in Fig 5(c), which is the same first step with example 1.

Seeing that line *P*_4_*P*_5_ and polyline ${P_{2}}^{\prime }P_{2}P_{3}$ do not intersect in Fig 5(d), the algorithm won’t go into the while-loop at line 12th in our algorithm, the way to acquire the implicit function of *α*_2_*α*_3_*α*_4_ is to apply the Boolean operation of *α*_2_*α*_3_ ∩ *α*_4_ because point *P*_5_ is inside *α*_3_. Region ① and region ② are inside the implicit function of *α*_2_*α*_3_, while region ② and region ③ are inside the implicit function of *α*_4_. Boolean operation of *α*_2_*α*_3_ ∩ *α*_4_ makes the region of ② inside the implicit function of *α*_2_*α*_3_*α*_4_. The implicit function of *α*_2_*α*_3_ is the solution of subproblem2, while the implicit function of *α*_4_ is the solution of subproblem1, making it possible to solve the whole problem.

In conclusion, the solution to the implicit function of this series of plane widgets can be expressed as follows:

$$\begin{array}{cc} \alpha_{1}\alpha_{2}\alpha_{3}\alpha_{4} & =\alpha_{1}\cup \left(\alpha_{2}\alpha_{3}\alpha_{4}\right)\\ & =\alpha_{1}\cup \left(\left(\alpha_{2}\alpha_{3}\right)\cap \alpha_{4}\right)\\ & =\alpha_{1}\cup \left(\left(\alpha_{2}\cup \alpha_{3}\right)\cap \alpha_{4}\right). \end{array}$$ (3)

**Fig 6:**
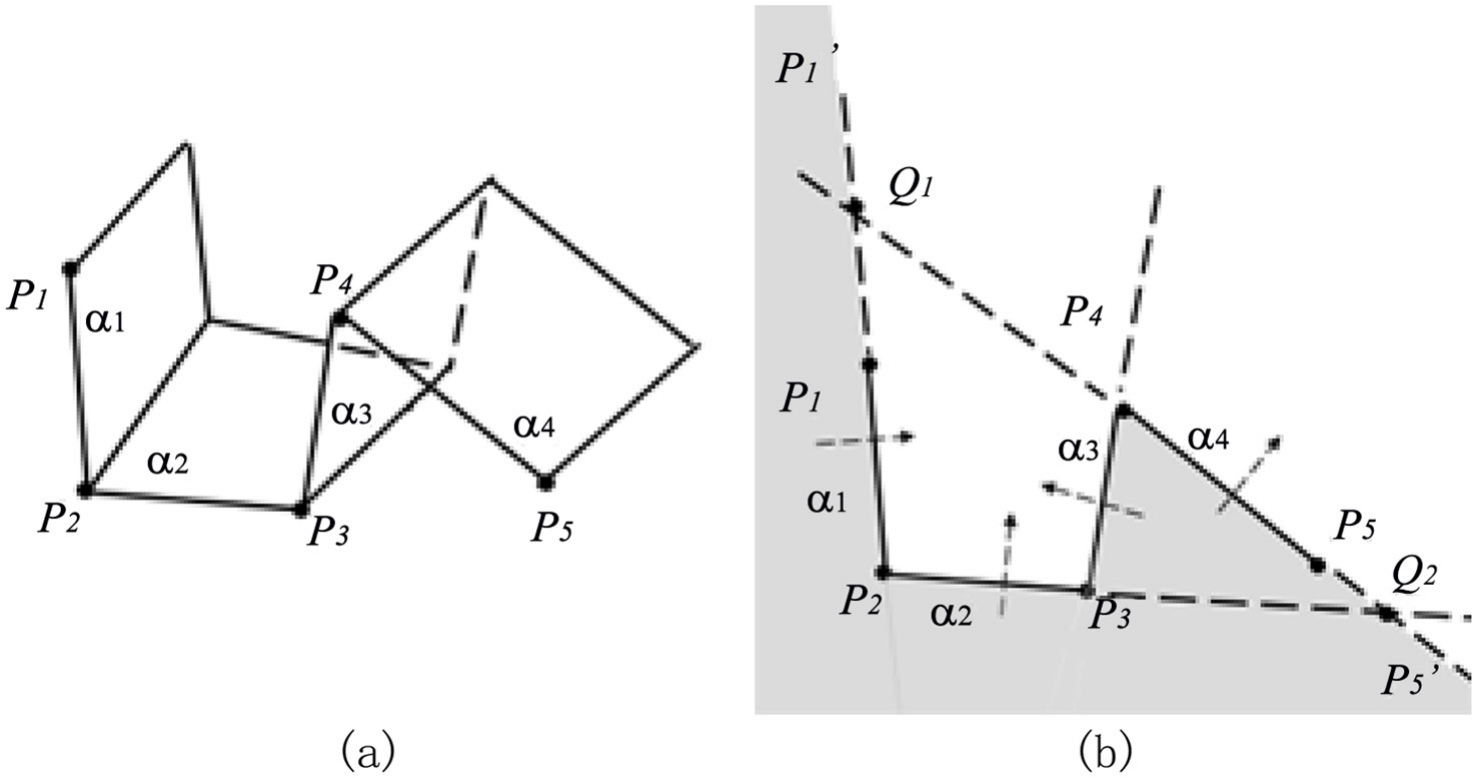
Implementation of algorithm for example3. (a)Clipping path with four plane widgets, (b)Transection view of clipping path.

For the last example in Fig 6(b), line *P*_4_*P*_5_ and polyline ${P_{1}}^{\prime }P_{1}P_{2}P_{3}$ at *Q*_1_ intersect. Then we decrease the value of *k* and find that line *P*_3_*P*_4_ and ray ${P_{1}}^{\prime }P_{1}P_{2}$ do not intersect. But it should be noticed that line *P*_2_*P*_3_ and polyline $P_{4}P_{5}{P_{5}}^{\prime }$ intersect at *Q*_2_, which satisfies the loop condition at line 29th in our algorithm. Thus, the value of *k* should still be decreased again and the implicit function of *α*_1_*α*_2_*α*_3_*α*_4_ equals to *α*_1_ ∪ *α*_2_*α*_3_*α*_4_. The implicit function of *α*_2_*α*_3_*α*_4_ is the same with that in example 2. So the implicit function of *α*_1_*α*_2_*α*_3_*α*_4_ is expressed as:

$$\begin{array}{cc} \alpha_{1}\alpha_{2}\alpha_{3}\alpha_{4} & =\alpha_{1}\cup \left(\alpha_{2}\alpha_{3}\alpha_{4}\right)\\ & =\alpha_{1}\cup \left(\left(\alpha_{2}\cup \alpha_{3}\right)\cap \alpha_{4}\right). \end{array}$$ (4)

Although the implicit function is the same with that in the Eq (2), the analysis of implementation process of the algorithm is actually different. Anyway, we get the result of the implicit function again successfully using our recursive algorithm.

These three simple examples illustrate the implementation of algorithm. In fact, the correctness of this algorithm has been widely tested, not merely the clipping path with four connected plane widgets, which will be discussed in the next section.

## Results

A module named SmartModelClip, served as computer-assisted surgical planning, is developed to clip the model on the basis of 3D Slicer and VTK using our recursive algorithm. The results of model clipping demonstrate the robustness of the algorithm.

### Module Development

3D Slicer(or Slicer) is a open-source and cross-platform(i.e., running on the system of Windows, Linux and Mac OS) software that processes the analysis and visualization of biomedical images [26]. The function of Slicer involves not only segmentation, registration and visualization of multimodal image data but also advanced image analysis algorithms for diffusion tensor imaging, functional magnetic resonance imaging and image-guided radiation therapy [27]. What’s more, Slicer allows loadable module that is developed by C++ or Python to extend its functionality. The hierarchy of Slicer illustrated in Fig 2 of [28] shows that VTK, and Qt are included for higher-level programmable functionality. VTK is powerful in medical image processing and data visualization because researchers can directly utilize its existing image and graphics algorithms to construct sub-systems to deal with image and graphics data, which prevents unnecessary repeated tasks [29]. Qt is a cross-platform application framework that is widely used because of its support for developing application software with a graphical user interface (GUI) [30], [31].

**Fig 7:**
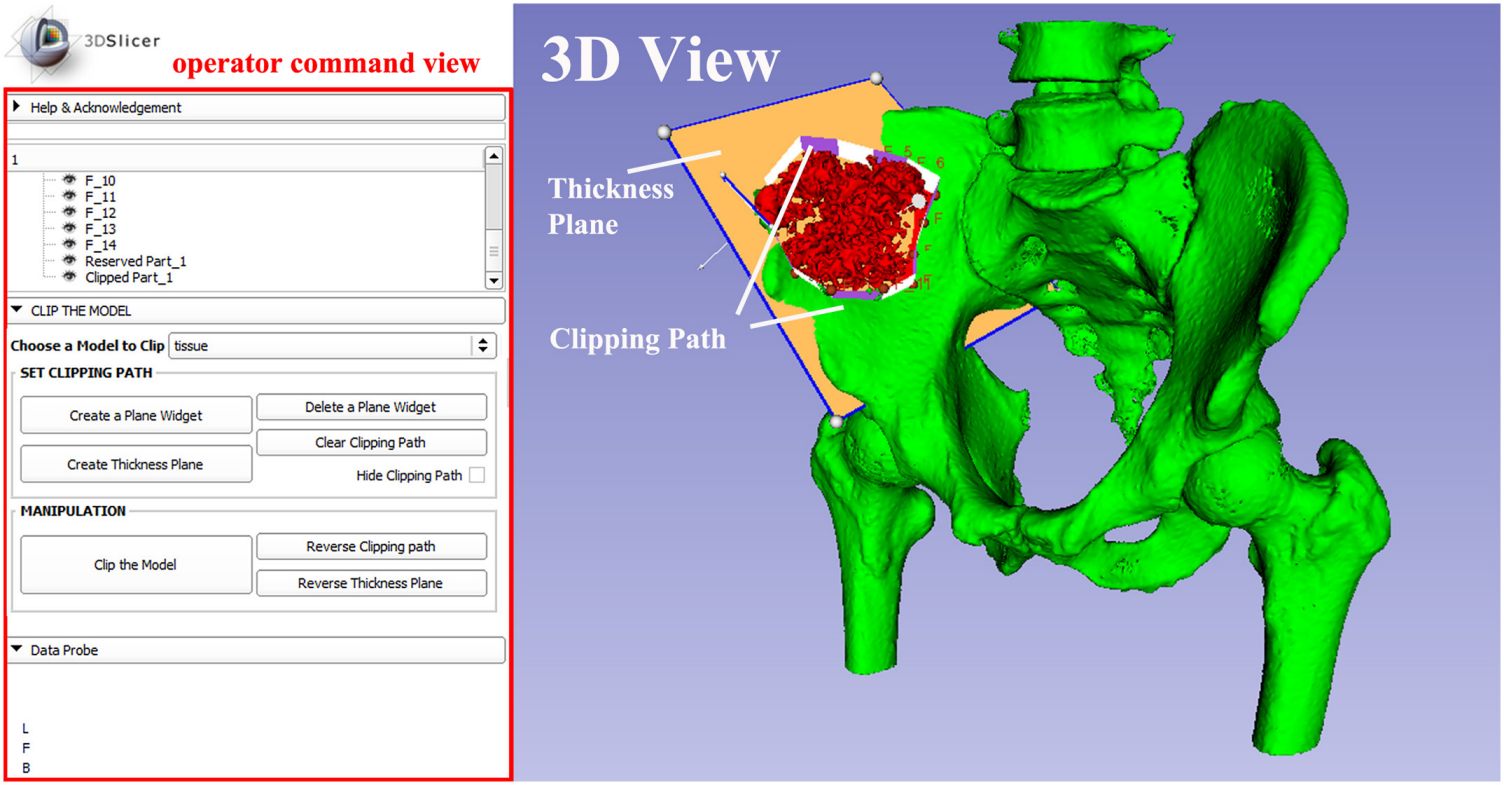
A snapshot of the module SmartModelClip. On the left is the operator command view that users can create and manipulate clipping path and thickness plane(i.e., they can create, hide and delete plane widgets). Users can also reverse the directions of the axes of the both clipping path and thickness plane widget. On the right is the scene that users can interact with the clipping path. They can specify the fiducial points that position the clipping path and modify the boundary of the clipping path by dragging the handles to obtain the desirable clipping path.

Our module is developed on the platform of 3D-Slicer, making use of VTK and Qt as visualization and GUI toolkits. Fig 7 shows the interface of our module, in which users can interact with the clipping path for computer-assisted surgical planning and do some manipulations to clip the model. Plane widgets are specified to the accurate position by fiducial points.

### Robustness Evaluation

#### Simple Clipping Path

**Fig 8:**
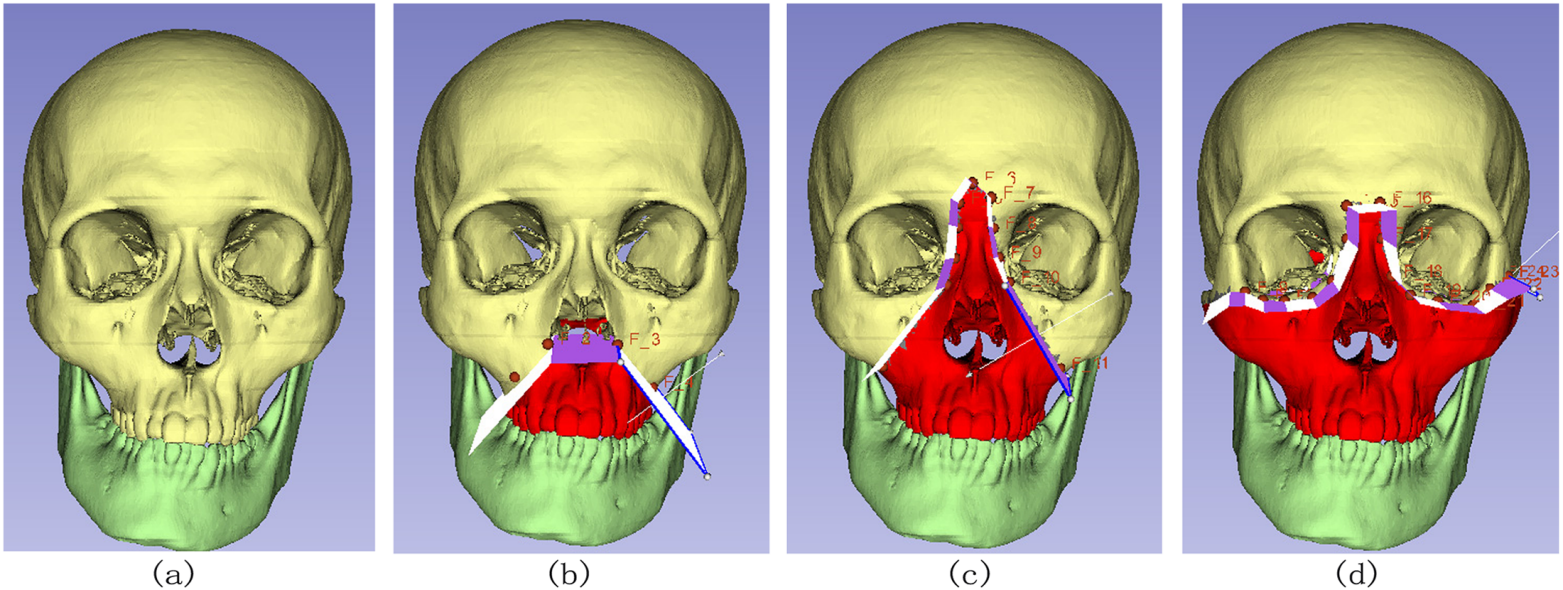
Test of simple clipping path. (a)Normal skull, (b)Clipping of Le Fort I fractures, (c)Clipping of Le Fort II fractures, (d)Clipping of Le Fort III fractures.

Le Fort fractures are common in facial trauma. Fig 8 shows three types of Le Fort fractures clipped by our module, demonstrating the robustness algorithm. These three types of Le Forts fracture do not have the explicit boundary for mesh segmentation, which makes our method of pre-surgical planning for model clipping weigh over mesh segmentation that can only separate the models with clear boundaries.

#### Complex Clipping Path

**Fig 9:**
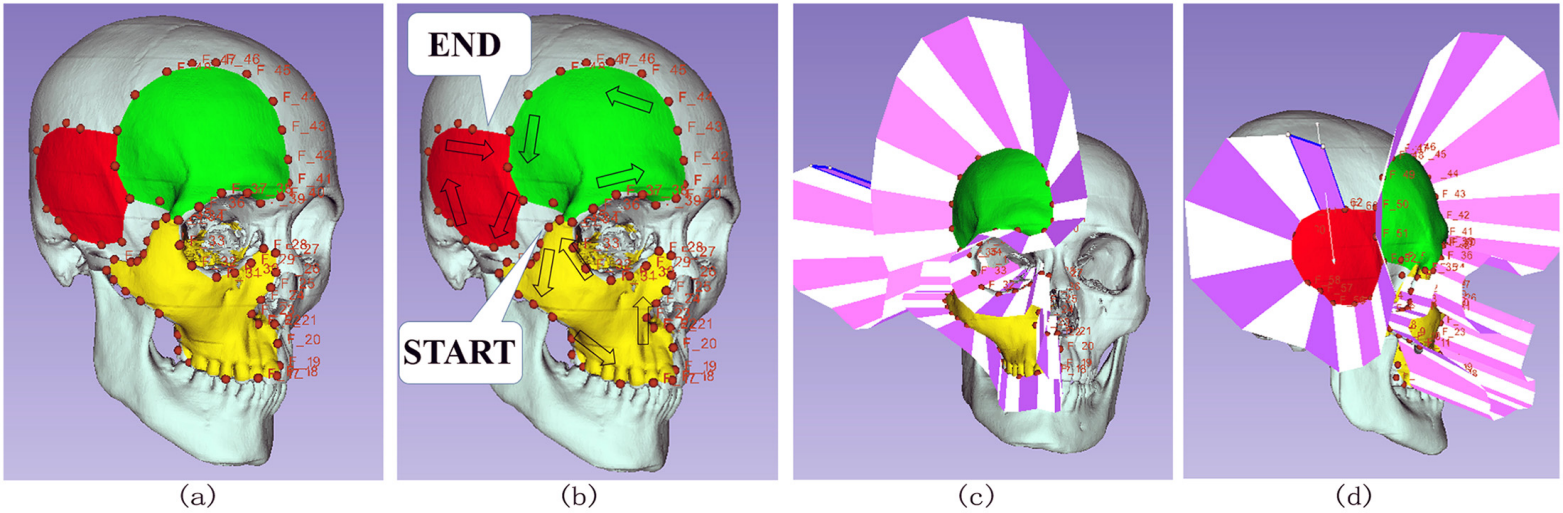
Test of complex clipping path. (a)Skull composed of maxilla and mandible, (b)Sequence of clipping path, (c)Front view of clipped skull, (d)Lateral view of clipped skull.

The clipping path can be more complex than the previous situation. In Fig 9(a), a clipping path splits the space into four regions and the skull is clipped into four parts along the clipping path.

The arrows in Fig 9(b) shows the sequence of the clipping path. We first specify the clipping path that encloses the yellow parts and cuts the yellow parts away from the light blue skull. Secondly, we extend the clipping path to enclose the green parts and clip the green parts away from the remaining skull in the first step. Finally, we extend the clipping path of the second step to enclose the red part and clip it away from the remaining skull in the second step. After all these steps, we obtain the clipped skulls in Fig 9(a). Fig 9(c) and 9(d) are front view and lateral view of our clipping result.

The clipping processes fit the reality in computer-assisted surgical planning because the yellow, green and red part of the model are clipped in sequence on a clipping path. What’s more, the boundary of the clipping path in Fig 9(d) is twisted from the front view to the lateral view, which makes it possible for the clipping path to make a turn in the 3D space. The clipping paths of other computer-assisted surgical planning softwares may not be twisted from one view to another, which makes our method for presurgical planning gain an advantage over other related softwares.

#### Thickness Specification

**Fig 10:**
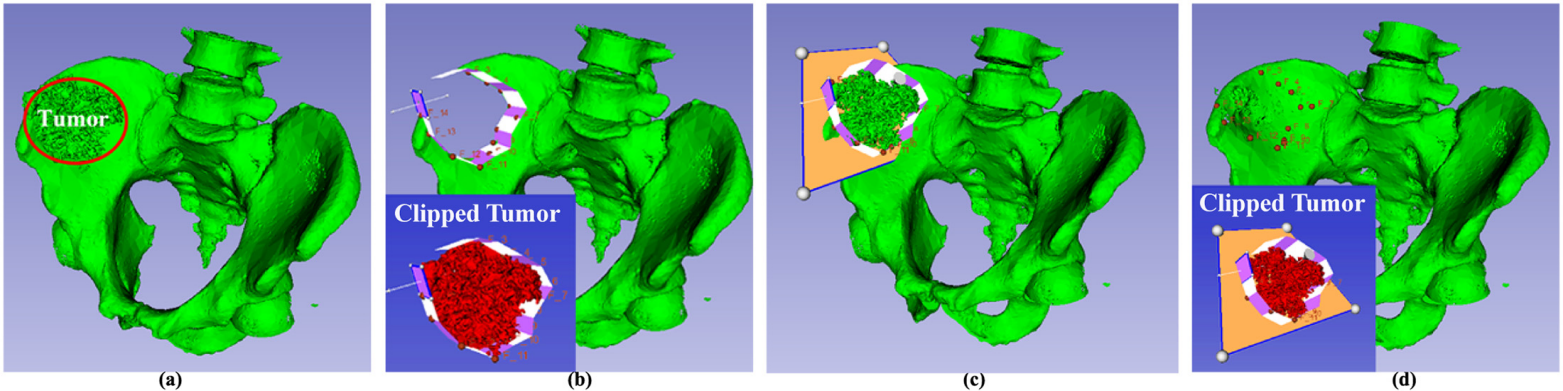
Presurgical planning for tumor removal. (a)Tumor required to be clipped, (b)Clipping of tumor without thickness plane, (c)Clipping path and thickness plane that enclose the tumor, (d)Result of presurgical planning for tumor removal by the clipping path in (c).

The thickness plane allows surgeons to clip model with certain thickness. Fig 10(a) is the pelvis with a tumor(circled by the red ellipse) and the task is to clip the tumor away from the pelvis. In Fig 10(b), the clipping path without thickness plane clips the tumor away from the pelvis, but the pelvis is actually cut through by the clipping path, which is the case of Magic RP referred in the section 1. This defect can be addressed by adding the thickness plane widget that specifies the thickness of the tumor that should be clipped away.

Fig 10(c) illustrates the clipping path with a thickness plane widget. The thickness of the clipped model is available through the Boolean intersection of the implicit function of the peripheral clipping path and that of the thickness plane widget. As the implicit function of clipping path and the thickness plane all have their own inside part and they can split the space into four parts, the button of ‘Reverse Clipping Path’ and ‘Reverse Thickness Plane’ serve to reverse the directions of the normal axes if the common inside part of the peripheral clipping path and the thickness plane widget does not enclose the tumor. If the direction of a plane widget is reversed, the inside part of the implicit function is reversed accordingly. Anyway, we can always clip the tumor with certain thickness in Fig 10(d) and the pelvis is not cut through, which is just what is desired in presurgical planning.

#### Limitations

1. Limitation in constructing the clipping path. Our recursive algorithm proves to be pretty reliable and robust for most experimental cases except for Moebius clipping path, however, the limitation may still exist in the construction of the clipping path. It’s noticed that our clipping algorithm is restrained to a series of plane widgets connected from the beginning to the end because the polyline referred in the section ‘Conversion of 3D Problem to 2D Problem’ is the judgment of the type of Boolean operations applied to each plane widget. The algorithm is able to obtain the implicit function of three connected plane widgets in Fig 2. However, in the real clinical practice, if the three plane widgets are adjacent, or perpendicular to each other, our clipping algorithm may fail due to the lack of judgment of the relative pose.
  Another limitation in the construction of the clipping path, is the case of complex clipping path in Fig 9. The clipping path is continuous, so if the user wants to clip several tumors at different locations at a time, for example, one tumor is at the front and the other tumor is at the back, it’s not easy to twist the clipping path. In such case, the users are suggested to construct another clipping path to clip the second tumor.
  For another case that our clipping algorithm may not work is the clipping path tangent to Moebius strip because Moebius strip is a surface with only one side. However, this kind of case may not appear in the computer-assisted surgical planning. So our algorithm still meets the need of the most clinical applications.
2. Limitation of thickness plane. As the back side of the tumor is unobservable, we only consider using one thickness planewidget to cut the tumor. But the curved surface of the back of the tumor to be clipped sometimes could be very complex, for example, it can be seen in the Fig 10(d) that some residues of the tumor remain on the pelvis. Future work would be considered to make the thickness plane widget more flexible, and more thickness plane widgets could be added to simulate the tumor clipping with no residues.

## Discussion

The time performance of the clipping algorithm for each case is first discussed and then the algorithm is evaluated based on the model clipping experiments, which demonstrates the time performance analysis of the clipping algorithm.

### Performance Of the Algorithm

The time performance of the recursive algorithm depends on the number of plane widgets and their relative poses. The best case runs as fast as the linear algorithm, while the worst case runs as slowly as the sub-quadratic algorithm. But the average case has the logarithmic time performance. For most cases of the computer-assisted surgical planning to remove the tumor, our clipping algorithm has the best time performance or the average-case performance.

#### Best-Case Performance

**Fig 11:**
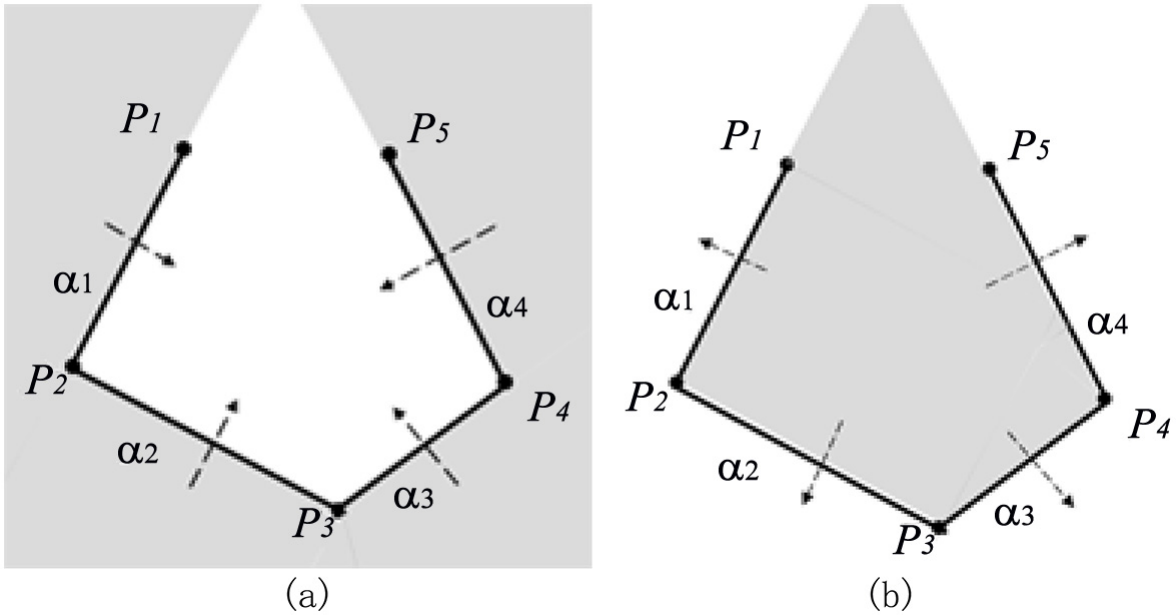
Two Cases of best time performance. Both of the shadow regions in Fig 11(*a*) and Fig 11(*b*) are inside the respective implicit functions.

The best case happens when the condition of the while-loop (from line 27th to line 30th) in our algorithm is not satisfied all through the stage of recursion. In that case, the recursive function will only apply Boolean operation of *α*_*j*_ ∪ *α*_*i*_*α*_*i*+1_⋯*α*_*j*−1_ or *α*_*j*_ ∩ *α*_*i*_*α*_*i*+1_⋯*α*_*j*−1_ in each recursion. Any case of the convex clipping path will have the best-case performance. Fig 11(a) and 11(b) best illustrates this, in which the implicit function of *α*_1_*α*_2_*α*_3_*α*_4_ can be expressed respectively by

$$\begin{array}{c} \alpha_{1}\alpha_{2}\alpha_{3}\alpha_{4}=\left(\left(\alpha_{1}\cup \alpha_{2}\right)\cup \alpha_{3}\right)\cup \alpha_{4} \end{array}$$ (5)

and

$$\begin{array}{c} \alpha_{1}\alpha_{2}\alpha_{3}\alpha_{4}=\left(\left(\alpha_{1}\cap \alpha_{2}\right)\cap \alpha_{3}\right)\cap \alpha_{4} \end{array}$$ (6)

In each case of Fig 11, the shaded region is inside the implicit function of *α*_1_*α*_2_*α*_3_*α*_4_.

As the problem is only decomposed into the subproblem1 and subproblem3 each time and the loop condition is never satisfied, the time complexity can be calculated by the sum of the time of solving subproblem1, subproblem3 and the time of the Boolean operation of the result of subproblem1 and subproblem3, which can be expressed in the following equation:

T(N)=T(1)+T(N-1)+1, (7)

where *T*(*N*) is the time of acquiring the implicit function of *N* plane series, *T*(*N* − 1) is the time of solving the subproblem3. Since the recursion just returns when *N* equals to 1 or 2, *T*(2)=*T*(1) = 1. So the above recurrence has the solution of

$$\begin{array}{cc} T(N) & =T(2)+2(N-2)\\ & =2N-3\\ & =O(N). \end{array}$$ (8)

We can see from the time complexity of the best case performance is the same with that of the linear algorithm.

#### Worst-Case Performance

The worst case behavior occurs in the situation that the condition inside the loop (at line 15) of the pseudo code is satisfied and the loop is executed at *j* − *i* − 1 times in each recursive call. Fig 4 illustrates this situation. The worst case applies Boolean operations from the last plane widget to the first plane widget, while the Boolean operation of best case goes the opposite, i.e., it applies the Boolean operation from the first plane widget to the last plane widget.

For the implicit function of *N* plane widgets, the time complexity can be calculated by the sum of three parts. The first part is the time to go through the loop for *N* − 1 − 1 times, the second part is the time to solve the subproblem3 and the last part is to take Boolean operations of the implicit functions. Thus the time complexity can be expressed in the equations of

$$\begin{array}{cc} \;T(N) & =(N-2)+T(1)+T(N-1)+1\\ & =T(N-1)+N \end{array}$$ (9)

with initial values of *T*(1) = *T*(2) = 1.

This recurrence has the solution

$$\begin{array}{cc} T(N) & =T\left(2\right)+\sum_{i=3}^{N}i\\ & =\frac{\left(N+3)(N-2\right)}{2}+1\\ & =O(N^{2}), \end{array}$$ (10)

the time complexity of which is quadratic. Although it’s quadratic time complexity, the occurrence possibility of the worst case is actually very low.

#### Average-Case Performance

As to the case of average time performance, we can consider the every possible looping times in the subproblem3 and take their average. If the possibility of the times that the loop will be executed is equal, then the average time to split the space with *N* plane widgets is the average time to execute the two recursive functions(at line 16th or line 19th in the algorithm) plus the time to do the loop and the time to apply the Boolean operations of the two recursive functions. The implicit function of *α*_1_ *α*_2_⋯*α*_*N*_ is decomposed into the Boolean operation of the implicit function of *α*_1_*α*_2_ ⋯ *α*_*i*_ and that of *α*_*i*+1_*α*_*i*+2_⋯*α*_*N*_. And the times to do the loop is *N* − (*i* + 1). So the time complexity of the average-case performance can be expressed as:

$$\begin{array}{c} T\left(N\right)=\frac{1}{N-1}\sum_{i=1}^{N-1}\left(T(i)+T(N-i)+(N-i-1)+1\right)\\ =N+\frac{2}{N-1}\sum_{i=1}^{N-1}T(i) \end{array}$$ (11)

with an initial value of *T*(1) = *T*(2) = 1.

The solution of this recurrence is

$$\frac{T(N)}{N}=1+2\left(InN+0.577-1\right).$$ (12)

The detail proof of this is in the appendix.

Consequently, the average time complexity of this algorithm is *O*(*N* log *N*).

In comparison with other polygon clipping algorithms, such as Sutherland-Hodgman Algorithm and Vatti clipping algorithm, our clipping algorithm has better time complexity in the computer-assisted surgical planning. For the general case, Sutherland-Hodgman Algorithm has the time complexity of *O*(M*N), in which *M* stands for the number of edges in the clipping polygon and *N* stands for the number of vertex pairs in the subject polygon. And Vatti clipping algorithm is twice as fast as Sutherland-Hodgman Algorithm and substantially faster if the algorithm is used for both clipping and filling [32]. Although our clipping algorithm has the same worst time performance of *O*(*N*^2^), the average time complexity of the algorithm is *O*(*N* log *N*) and the best time complexity of *O*(*N*). In fact, as the clipping path is defined manually according to the boundary of something like tumor, the number of the clipping path widgets would not be very large(let’s say 15 or 30). In that case, the time complexity of our algorithm is approximately linear, making it possible to separate the 3D space along the clipping path in a very short time.

### Time Performance Evaluation and Comparison

#### Evaluation of our method

The time to clip the model is actually composed of two parts: the first part is the time of acquiring the implicit function of the clipping path by our algorithm and the second part is the time to clip the triangles of the model by the class of ‘vtkClipPolydata’ in VTK. These two parts of time are tested respectively. The execution time of the recursive algorithm is tested using different clipping paths that have different number of plane widgets, while the second part of time performance is tested by clipping the models that have different number of triangles using the same clipping path. The experiments are tested on the laptop with the Intel CPU T1400 @1.73GHz and 2GB DDR2 RAM.

**Fig 12:**
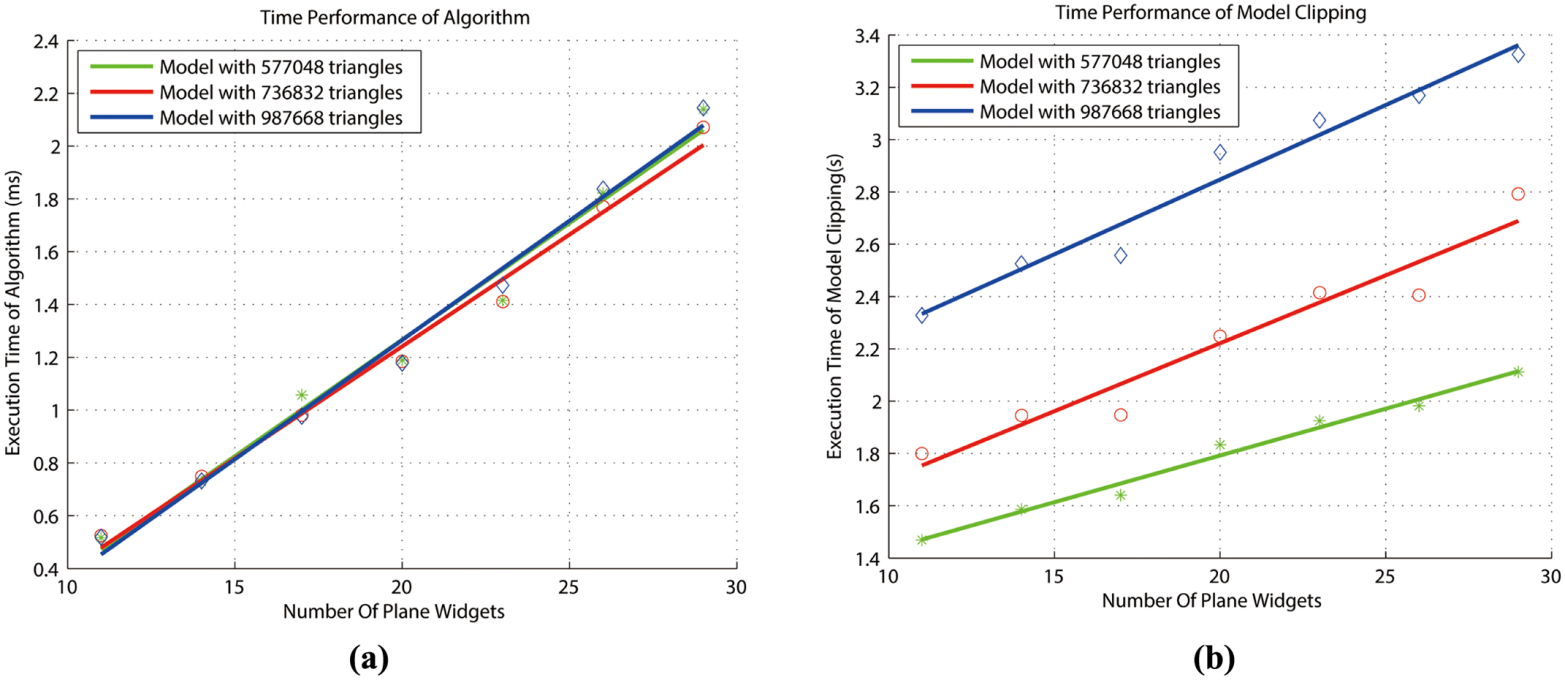
Time performance. (a)Time performance of executing the recursive algorithm. (b)Time performance of model clipping.

We evaluate the time performance by clipping the tumor in Fig 10(a). The model can be reconstructed by Magic RP with different number of triangles, so we generate three models that have 577,048, 736,832 and 987,668 triangles respectively. We first create 29 plane widgets that enclose the tumor as is the case in Fig 10(c) and then the three models are clipped by this clipping path. As these plane widgets are fairly enough to form the clipping path, we decrease the number of plane widgets gradually by deleting some of the fiducial points and form a new clipping path. Then we clip the three models again by the new generated clipping path. After some tests, we obtain the time to acquire the implicit function and the time to clip the model in Fig 12. The results show that our algorithm is highly efficient because the time to acquire the implicit function is in the order of microseconds, and the time to clip the triangles is in the order of seconds for the model that has up to one million triangles. Also the time to execute the algorithm is almost in direct proportion to the number of plane widgets because the boundary of the clipping path that encloses the tumor is convex-like, which is congruent to the best-case time performance analyzed in this section. The time performance of acquiring the implicit function has nothing to do with the models with different number of triangles. But the time to clip the model is related to both the number of triangles and the number of plane widgets. When the number of triangles increases, the time to clip the model increases accordingly. And for the same model, the time to clip the triangles also increases proportionally with the number of plane widgets. In a word, the results show the correct analysis of the time performance of the recursive algorithm and the presurgical planning method is highly efficient.

#### Comparison with other methods

In terms of the segmentation time, our method has an advantage over Random Walks Algorithm and fitting primitives Algorithm. Since the time to separate the model relates both to the computation speed of CPU and the complexity of the model, we only make the rough comparison of each method’s average segmentation speed, in which the CPU speed is almost the same with each other.

The timing performance of various models mentioned in TABLE 1 of [12] shows that the segmentation time with Watershed preprocessing step is approximately linear to the number of vertices. Although the models being clipped are different and the actual number of vertices being segmented is much less than the total number of vertices, it seems that the segmentation time is linear to the total number of vertices. So the average clipping speed of different models in the [12] is about 248 thousand vertices/second, and the average segmentation speed referred in the [13] is about 13 thousand triangles/second. Compared with these algorithms, our method has a faster segmentation speed of about 290 thousand triangles/second. In fact, the segmentation speed is not the only advantage of our method, and what matters most is that it could be used in interactive surgical planning, which is beyond the ability of the other segmentation methods.

## Conclusions

In this paper, a recursive algorithm is developed for clipping models based on the Boolean combination of the implicit functions of the series of the connected plane widgets. The problem of splitting 3D space by clipping path is degraded into the equivalent problem of splitting 2D space by a polyline. The recursive algorithm then decomposes the problem of splitting 2D space into three subproblems that makes the whole problem easy to solve. Three examples of four connected plane widgets illustrates the implementation process of the algorithm.

A module named SmartModelClip is developed for computer-assisted surgical planning using our recursive algorithm. Three experiments of model clipping for presurgical planning has successfully demonstrated the robustness of the module: (1)presurgical planning with simple clipping path for clipping Le Forts fractures, (2)presurgical planning with complex clipping path that can be twisted from one view to another for clipping the model into several parts, (3)presurgical planning with thickness plane for tumor removal. The limitations in the construction of the clipping path and thickness plane widget are discussed in details.

After that, the time performance of the algorithm is analyzed. The time performance is relevant to the poses of the series of plane widgets. The time complexity of the best-case performance is *O*(*N*) and the time complexity of the worst-case performance is *O*(*N*^2^), while the time complexity of the average-case performance is *O*(*N* log *N*). The algorithm tends to have linear time performance when the removed part is enclosed by the clipping path. Rough comparison with other polygon clipping algorithms shows the efficiency of our algorithm in the presurgical planning.

The time performance of model clipping and the execution time of our algorithm to acquire the implicit function of clipping path are both evaluated highly efficient in two tests: (1)the test of time to clip different models that have different number of triangles with the same clipping path, (2)the test of time to clip the same model with the clipping paths that have different number of plane widgets. Compared with other segmentation methods, our method has an advantage over these methods in terms of the average segmentation time.

## Appendix

### Proof of the Average-case Time Performance

From Eq (11), we know the average-case time complexity can be expressed in the following recurrence:

$$\left\{\;\begin{array}{c} T\left(N\right)=N+\frac{2}{N-1}\sum_{i=1}^{N-1}T(i)\\ T(1)=T(2)=1. \end{array}\right.$$ (13)

We want to prove that the Expression (13) has the time complexity of *O*(*N* log *N*).

Multiply (*N* − 1) on both sides of the Eq (13), we obtain

$$\left(N-1\right)T\left(N\right)=N\left(N-1\right)+2\sum_{i=1}^{N-1}T(i).$$ (14)

Substitute *N* with *N* − 1, we obtain the recurrence of

$$\left(N-2\right)T\left(N-1\right)=\left(N-1\right)\left(N-2\right)+2\sum_{i=1}^{N-2}T(i).$$ (15)

Subtract Eqs (15) from (14), the result is

(N-1)T(N)=N·T(N-1)+2(N-1). (16)

Divide both sides of the Eq (16) by *N*(*N* − 1), we obtain

$$\frac{T(N)}{N}=\frac{T(N-1)}{N-1}+\frac{2}{N}.$$ (17)

By iterating the Eq (17), we obtain the group of equations

$$\begin{array}{c} \left\{\begin{array}{cccccc} & \frac{T(N)}{N} & = & \frac{T(N-1)}{N-1} & + & \frac{2}{N}\\ & \frac{T(N-1)}{N-1} & = & \frac{T(N-2)}{N-2} & + & \frac{2}{N-1}\\ & \; & \vdots \\ & \frac{T(2)}{2} & = & \frac{T(1)}{1} & + & \frac{2}{2}. \end{array}\right. \end{array}$$ (18)

Sum up all the equations of Eq (18), we obtain

$$\begin{array}{cc} \frac{T(N)}{N} & =T\left(1\right)+2c\sum_{i=2}^{N}\frac{1}{i}\\ & =c+2c(In(N)+0.577-1)\\ & =O(\log N). \end{array}$$ (19)

Consequently, the average-case time complexity is

T(N)=O(NlogN). (20)
